# Different contractility modes control cell escape from multicellular spheroids and tumor explants

**DOI:** 10.1063/5.0188186

**Published:** 2024-05-07

**Authors:** Eliane Blauth, Steffen Grosser, Frank Sauer, Mario Merkel, Hans Kubitschke, Enrico Warmt, Erik W. Morawetz, Philip Friedrich, Benjamin Wolf, Susanne Briest, Grit Gesine Ruth Hiller, Lars-Christian Horn, Bahriye Aktas, Josef A. Käs

**Affiliations:** 1 Peter Debye Institute for Soft Matter Physics, Leipzig University, Linnéstraße 5, 04103 Leipzig, Germany; 2 Department of Gynecology, Leipzig University Hospital, 04103 Leipzig, Germany; 3 Division of Breast, Urogenital and Perinatal Pathology, University Hospital Leipzig, Leipzig, Germany; 4 Institute for Bioengineering of Catalonia, The Barcelona Institute for Science and Technology, Barcelona 08028, Spain

## Abstract

Cells can adapt their active contractile properties to switch between dynamical migratory states and static homeostasis. Collective tissue surface tension, generated among others by the cortical contractility of single cells, can keep cell clusters compact, while a more bipolar, anisotropic contractility is predominantly used by mesenchymal cells to pull themselves into the extracellular matrix (ECM). Here, we investigate how these two contractility modes relate to cancer cell escape into the ECM. We compare multicellular spheroids from a panel of breast cancer cell lines with primary tumor explants from breast and cervical cancer patients by measuring matrix contraction and cellular spreading into ECM mimicking collagen matrices. Our results in spheroids suggest that tumor aggressiveness is associated with elevated contractile traction and reduced active tissue surface tension, allowing cancer cell escape. We show that it is not a binary switch but rather the interplay between these two contractility modes that is essential during this process. We provide further evidence in patient-derived tumor explants that these two contractility modes impact cancer cells' ability to leave cell clusters within a primary tumor. Our results indicate that cellular contractility is an essential factor during the formation of metastases and thus may be suitable as a prognostic criterion for the assessment of tumor aggressiveness.

## INTRODUCTION

The spreading of cancer cells is a crucial step during tumor progression. As a result, cancer becomes a systemic disease, which is responsible for the majority of cancer-associated deaths.^1–3^ Thus, it is critical to understand how cancer cells can leave the primary tumor at the beginning of the metastatic cascade.^4,5^ A main initial prerequisite is the interaction of the cancer cells and cancer cell clusters with the extracellular matrix (ECM) surrounding the primary tumor mass. To form metastases, the originally non-motile cells must become motile and escape from the initial cell cluster into the ECM. In a densely packed environment with no free space like cancer cell clusters, this is achieved by an unjamming transition.^6–8^ The cells elongate their shapes to move by each other and exert traction forces onto the ECM to leave the cell aggregates, thereby restructuring the ECM network.^8–10^ This leads to a feedback loop, where cells pull on the ECM fibers, which in turn reorientate and might guide the cells away from them.^11–13^ For efficient cell invasion, the actin filaments assemble into myosin-rich stress fibers that are embedded in the actin cortex,^14^ and the cells elongate and adapt a bipolar, directed contractility mode, thereby pulling on the ECM.^15–17^ This migration pattern is typical for cells with a mesenchymal phenotype.^18^ On the contrary, cells with an epithelial phenotype build less stress fibers and form a cortical actin network as their dominant actin structure, creating a more isotropic contraction pattern that can even be seen in suspended cells.^15,19^ Moreover, cells with high cortical contractility can even form a collective actin rim around cell clusters, which generates a high tissue surface tension in agreement with the extended differential adhesion hypothesis.^19^ This raises the question of how these different contractility modes contribute to cancer cell escape and how they are related to tumor aggressiveness.

In the present study, a three-dimensional assay is used to probe the interaction of multicellular spheroids and patient-derived tumor explants on three-dimensional collagen type I networks. Collagen type I is the main constituent of fibrous mammalian ECM, making it a good trade-off between a model system and the real tumor microenvironment.^20^ The assay presented here is highly versatile and easy to deploy, yet able to assess cell escape and traction force generation, which is then related to the two different contractile modes in a more realistic scenario than in two-dimensional traction force assays. First, spheroids formed from a well-established breast cancer cell panel are used, namely, the non-tumorous MCF-10A cell line as a control and the MDA-MB-436 and MDA-MB-231 cell line as model for moderately invasive and highly invasive tumor cells, to correlate their aggressiveness with these parameters. The invasiveness increases with a change from a more epithelial to a more mesenchymal phenotype.^16,21^ The dynamic restructuring of the collagen network is analyzed and correlated with the spreading behavior of multicellular spheroids. In the next step, patient-derived tumor explants are used. By studying how cells can escape from cell aggregates surrounded by ECM, the present work provides a deeper understanding of the early events of the metastatic cascade, which may lead to novel prognostic criteria to predict early metastatic risk.

## RESULTS

### Collagen displacements and cell escape from multicellular spheroids correlate with their aggressiveness

To probe how the aggressiveness of cancer cells is reflected in cell escape from the initial cell cluster, i.e., multicellular spheroids, and in the generation of traction forces, an established breast cancer cell panel covering a range of invasive behaviors is used.^10,19^ The multicellular spheroids are placed on top of already polymerized collagen networks. Thus, we study the spreading of cancer cells on and into the collagen network, i.e., wetting on and cell invasion into the fibrous matrix. This can occur collectively as a cell front or individually by single cell escape. The ease of use of the assay permits large numbers of experiments with a high reproducibility. Recent publications have demonstrated the relevance of cell wetting of collagen sheets for cancer research.^22–24^ Real tumors are not embedded in pure collagen but surrounded by a complex microenvironment^25–27^ and cancer cells often migrate along boundaries.^12,22^ The displacements of the collagen network underneath the spheroids serve as a measure of the cell-ECM interaction and the cell spreading as a measure of cell escape. The displacements are detected in the bright-field images with a PIV (particle image velocimetry) algorithm.

**FIG. 1.**
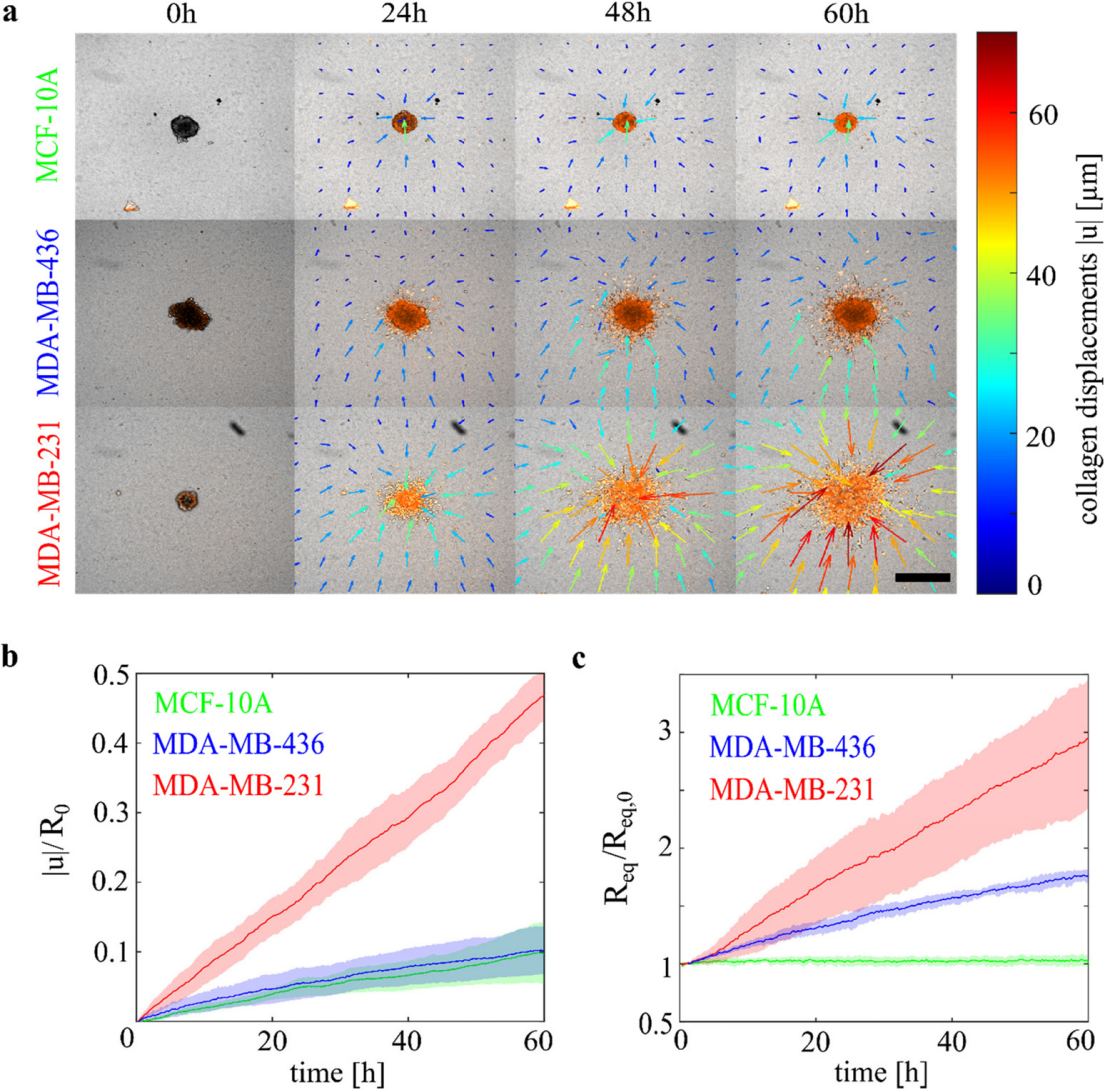
Spheroids made from a panel of breast cancer cell lines can be distinguished by distinct collagen displacement and cell escape. (a) Temporal evolution of different multicellular spheroids on 1.5 g/l collagen. The color and length of the arrows indicate the strength and direction of the unnormalized collagen displacements (see colorbar on the right). Cell nuclei are stained with SiR-DNA. Scalebar: 500 *μ*m. (b) Normalized mean collagen displacement |u|/R_0_ and 95% confidence interval (shaded area) over time. The displacements are averaged over the field of view of 1000 × 800 *μ*m^2^ and normalized to the initial spheroid radius, which is in the range of 100 *μ*m for all cell lines. The highly invasive MDA-MB-231 cells create the highest displacements, followed by MDA-MB-436 and then MCF-10A spheroids. N = 5 for each cell line. The local distribution around the spheroids of the displacement curves can be found in Fig. S3. (c) Cell escape characterized by the equivalent, normalized spreading radius R_eq_/R_eq,0_, shading corresponds to 95% confidence interval. The epithelial MCF-10A spheroids stay together as a compact cell cluster. MDA-MB-436 cells migrate moderately into the collagen, while MDA-MB-231 cells invade the collagen network extensively. N = 5 for each cell line.

 Spheroids from all three cell lines exert traction forces on the collagen matrix toward the spheroids. Figure 1(a) shows a time series of their pulling and invasion behavior on 1.5 g/l collagen networks. The arrows indicate the direction of the collagen displacements, and their strength is color-coded. A quantification of the collagen displacements, represented as the magnitude of the cumulative displacements |u|, can be found in Fig. 1(b). Here, the collagen displacements are normalized to the corresponding initial spheroid radius R_0_ as larger spheroids generate larger displacements.^28^ The metastatic MDA-MB-231 spheroids cause the largest collagen displacements, pulling the strongest on the ECM and generating the highest traction forces. The less invasive MDA-MB-436 spheroids displace the collagen fibers significantly less. After 60 h, the MDA-MB-436 spheroids created, on average, cumulative displacements on the order of 10% of their initial size compared to 50% in the case of the MDA-MB-231 spheroids. For these two cell lines, a correlation between the degree of collagen displacements and cancer aggressiveness can be observed. However, the normalized collagen displacements generated by the epithelial MCF-10A spheroids are in a similar range to those created by the cancerous MDA-MB-436 spheroids. At the end of the observation time, the MCF-10A and the MDA-MB-436 spheroids show nearly the same mean value, and the confidence intervals highly overlap [see Fig. 1(b)].

The presence of collagen displacements for all three spheroid types demonstrates that all of them interact with the ECM which lead to local stiffening around the spheroid (supplementary material Fig. S5). However, the displacement curves of the non-tumorous MCF-10A spheroids and the cancerous MDA-MB-436 spheroids do not stratify, which is why the cell spreading is used as a second parameter to discriminate cancer aggressiveness on a more differentiated level. It is quantified by the equivalent spreading radius, normalized to the initial radius,

$$R_{\mathrm{eq},\;\text{norm}}=\frac{R_{\mathrm{eq}}}{R_{\mathrm{eq},0}},$$

where R_eq,0_ denotes the initial spheroid radius at the start of the measurement. The equivalent radius R_eq_ is calculated at each time step from the area A that is covered by the fluorescent-labeled cells via

$$R_{\mathrm{eq}}=\sqrt{\frac{A}{\pi }}.$$

In contrast to the collagen displacements, the spreading behavior of the MCF-10A cells is clearly distinct from the MDA-MB-436 cells. The MCF-10A spheroids stay together as a compact cell mass during the whole observation time without a notable number of cells leaving the spheroid [see Figs. 1(a)–1(c)]. The cancerous MDA-MB-436 cells continuously leave the spheroid, while some cells remain as a compact cluster in the middle. It should be noted that the initial MDA-MB-231 spheroid was built with additional Matrigel^TM^ as MDA-MB-231 cells only form loose aggregates without it. The cells escape extensively from the initial spheroid, represented by the steep increase in the red curve in Fig. 1(c). An additional analysis of the angular direction of the collagen displacement with respect to the local cell spreading has not produced consistent results. We could not find a strong correlation between both parameters (see supplementary material and Fig. S3).

All spheroid types contract the collagen network but the contractile mechanisms seem to differ and result in different spreading behaviors which correlate well with the aggressiveness of the used cell lines. Quantitatively similar results were obtained for 3.0 g/l collagen networks (see Fig. S1). Nevertheless, the observed effects, especially the collagen displacements, are more pronounced for the softer collagen network and more reliable to detect. Thus, we focus on 1.5 g/l collagen networks for the experiments with primary tumor explants.

### Collective actin cytoskeleton acts as an energy barrier for cell escape

Previous studies showed that in ECM networks, epithelial cells display a strongly reduced directed stress-fiber based contractility compared to mesenchymal cells.^15,29–31^ However, epithelial cells can also contract their actin cortex even in the absence of focal adhesions.^19,32,33^ This so-called cortical contractility (σ_int_) of single (suspended) cells is quantified here with the optical stretcher. The optical stretcher (OS) is a dual laser beam trap, where the cells are deformed along the laser axis. It allows us to measure the active and passive biomechanical properties of cells without any influence of a substrate, which has been described in detail in Refs. 19, and 34. The active cell response counteracts the external stress applied with OS. It is quantified by fitting an adapted standard linear solid model to the deformation curve of each single cell. The model consists of a standard Kelvin–Voigt model^35,36^ with an additional spring in series and an additional active term that represents the active cortical contractility. The peak stresses exerted on the cells are on the order of 1 Pa, resulting in cell deformations of few percentages. The cells respond with forces in the same order of magnitude. Thus, the amplitude of these values should not be directly compared to the ones of adherent cells on substrates which are orders of magnitude higher.^31,37,38^

**FIG. 2.**
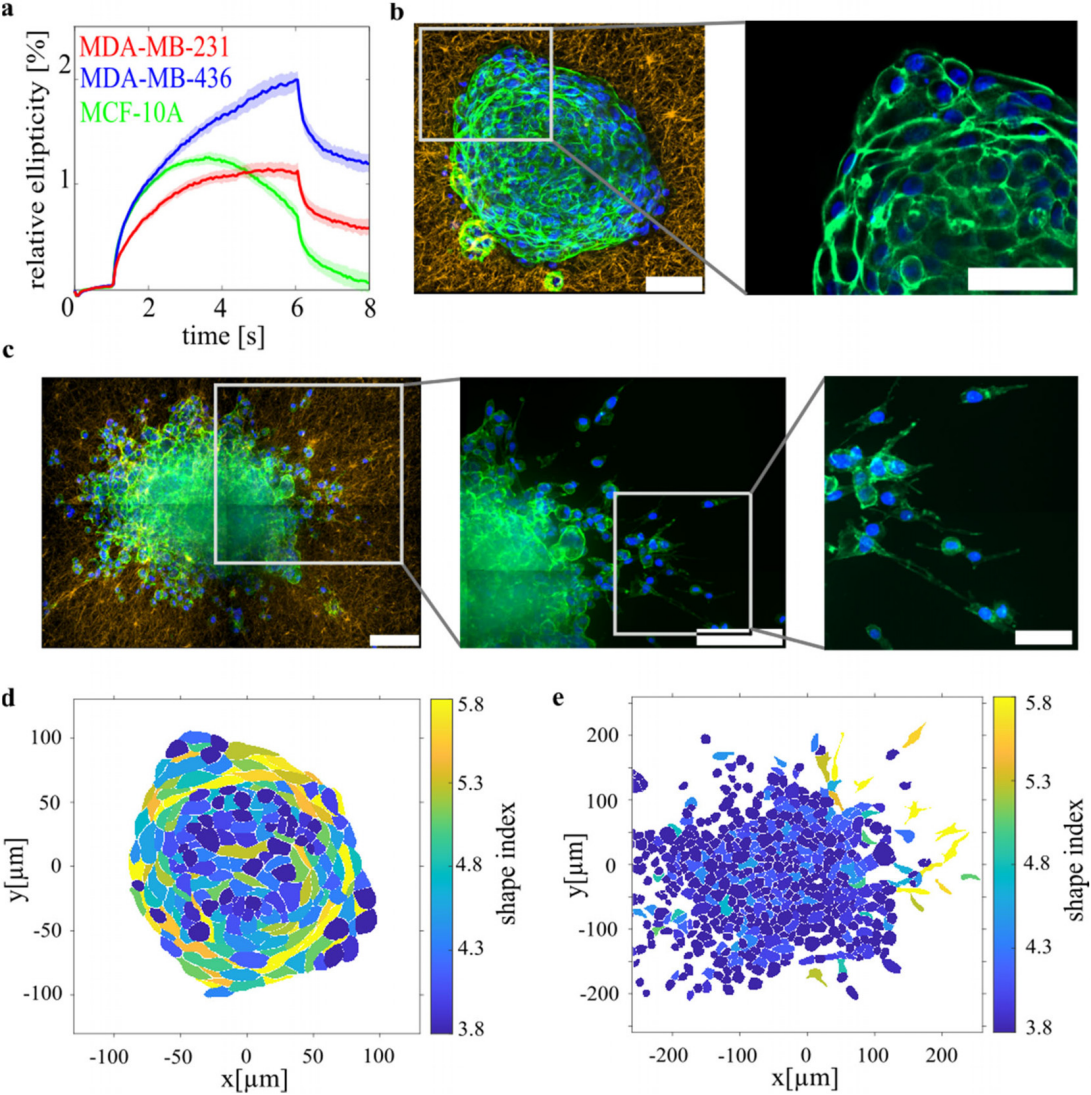
Relation between cortical single-cell contractility and interaction between ECM and multicellular spheroids. (a) Relative deformation of single cells in suspension against external pulling forces in the optical stretcher. The epithelial MCF-10A cells start to contract themselves after 2 s of stretching time and oppose the external forces. Neither of the mesenchymal cell lines (MDA-MB-436, MDA-MB-231) shows such a strong cortical contractile behavior (b) Left: MCF-10A spheroid stained for actin (green) and nuclei (blue) on a 1.5 g/l collagen substrate (orange). Right: confocal section through the middle of the same spheroid. The cortical actin rims of the individual cells are clearly connected and form a smooth collective shell surrounding the spheroid. The stretched, elongated cells aligned along the spheroid boundary indicate a high tissue surface tension. Scale bar: 50 *μ*m. (c) Confocal images (zoomed-in sections from left to right) of an MDA-MB-436 spheroid on a collagen matrix. The spheroid surface is rough pointing toward a low tissue surface tension. Single cells detach from the cell clusters, showing elongated actin structures caused by stress fibers supporting directed traction force generation. Scale bars: 100, 100, and 20 *μ*m. Note that the collagen structure in all images shown here is not representative as we used small cutouts around the spheroids to better image the actin structures of the cells. This may have disturbed the stress profile and orientation matrix caused by the interaction with the spheroid (for further details see Methods section) (d) 2D cell shapes analysis in the equatorial plane of the MCF-10A spheroid shown in (b). Cells in the middle are round while the ones at the boundary are tangentially elongated and generate a smooth boundary, which indicates a high tissue surface tension. (e) 2D cell shape analysis of the MDA-MB-436 spheroid shown in c. The cells are roundish throughout the whole spheroid and especially also at the boundary, indicating a low to vanishing tissue surface tension.

MCF-10A cells start actively contracting against the exerted stretching force [Fig. 2(a)] with a median contractile stress of σ_int_ = 1.09 (0.36 1.89) Pa, the numbers in brackets correspond to the quantile values. Since cells in suspension have no stress fibers, this contractility is mainly generated by the thin actin cortex located directly under the cell membrane. In suspended cell clusters, epithelial cells form a collective actin rim at the cluster boundary, generating a smooth surface, which indicates a high tissue surface tension.^19^ To test if epithelial cells keep this collective actin structure when put on collagen matrices, an MCF-10A and an MDA-MB-436 spheroid are fixed and stained after they interacted 72 h with the collagen networks. To better image the actin structures, we punched out small cutouts around the spheroids and flipped the sample. However, the punching out may have disturbed the stress profile and orientation of the collagen matrix caused by the interactions with the spheroid (for further details see Methods section). Thus, we have been careful in making statements about the effects on the collagen matrix. Here, we focus on the role of cortical vs stress fiber-based cell contractility with respect to the interactions of multicellular spheroids with the surrounding collagen matrix.

For the MCF-10A spheroids, the cells show distinct cortical actin structures that can form intercellular connects at the spheroid boundary. The cells at the boundary pull on each other, which becomes visible by the elongated cell shapes, and results in a smooth spheroid contour [Fig. 2(b)]. The resulting tissue surface tension holds the cell cells back in the spheroid. The presence of highly elongated cells at the boundary is confirmed by an analysis of the 2D cell shapes in the equatorial plane of the spheroid [Fig. 2(d)]. Despite that collagen is also an attractive substrate for the cells, visible by the pulling forces generated by the cells, MCF-10A spheroids with their roundish surface, smooth boundary, and their elongated cell shapes along the spheroid boundary have all the signatures of a sharp boundary between cells and collagen that requires a high tissue surface tension.^6,19,39^

With respect to cortical cell contractility and cell escape from multicellular spheroids, we have previously proven that cortical contractility strongly contributes to tissue surface tension by forming a collective contractile shell around the spheroid that holds cells back. This can be seen since an inhibition of the actin myosin complex, which significantly decreases cortical contractility of single cells, lowers the bulk modulus,^19,40^ i.e., surface tension, of multicellular spheroids leading to a visibly rougher spheroid surface. In contrast, MDA-MB-436 spheroids do not form such a collective contractile actin shell. The actin staining in Fig. 2(c) shows a representative MDA-MB-436 spheroid. While the cortical actin is reduced with respect to MCF10A cells, the cells in the spheroid are still connected by cell–cell adhesion, but the surface of the cluster is rougher and more irregularly formed than in the epithelial spheroids. The cell shapes are more round at the boundary, and no tangentially elongated cells are seen, which results in a rough spheroid surface [Fig. 2(e)]. The weak cortical actin structures and the rough spheroid surface agree with our measurements of a weak cortical contractility observed in the optical stretcher. These findings show that MDA-MB-436 spheroids exhibit a lower tissue surface tension.^19^ Thus, single cells or cell strands can escape more easily into the surrounding collagen matrix. Less adhesive cells invade as single cells, while more adhesive cells move collectively.^41^ Both can be observed in the case of the MDA-MB-436 cells, as the cells within one cell line still show a high heterogeneity,^42,43^ resulting in different adhesiveness and thus invasion behavior. The absence of a contractile actin cortex does not mean that the cells do not contract at all. The invading cells form directed contractile actin structures, reminiscent of actin stress fibers, to migrate and pull on the collagen network [Fig. 2(c)]. MDA-MB-436 cells that migrate into the surrounding matrix are clearly radially elongated and contract the substrate, which requires an anisotropic, force dipole-like contractility mode, predominantly generated by stress-fibers.^15^ The highly invasive MDA-MB-231 spheroids create the strongest collagen displacements and show a higher cortical contractility in the OS than the MDA-MB-436 cells [σ_int,231_ = 0.39 (0 1.34) Pa > σ_int,436_ = 0.15 (0 0.84) Pa]. In this highly invasive cell line, both contractility modes are present on a stronger level, than in the moderately invasive MDA-MB-436 cells. This suggests that it is not an either cortical or stress fiber contractility question, but more a question of the interplay and which contractility mode dominates over the other.

Our results add to our previous findings that epithelial and mesenchymal cells display different contractility modes relying either on the actin cortex or on stress fibers, respectively. Both cell types can exert forces on the collagen matrix but lead to distinctively different migration behaviors in the ECM. Epithelial cells predominately use the actin cortex to contract, which results in a collective rim and strong inter-cellular interaction, which holds the cells together and back from leaving the spheroid. Mesenchymal cells, in contrast, favor contractile stress fibers that generate a directed pulling force between cells and ECM, which provides the traction forces that cells can leave the spheroids.

### Collagen displacements and cell escape in primary tumor explants indicate their aggressiveness

**FIG. 3.**
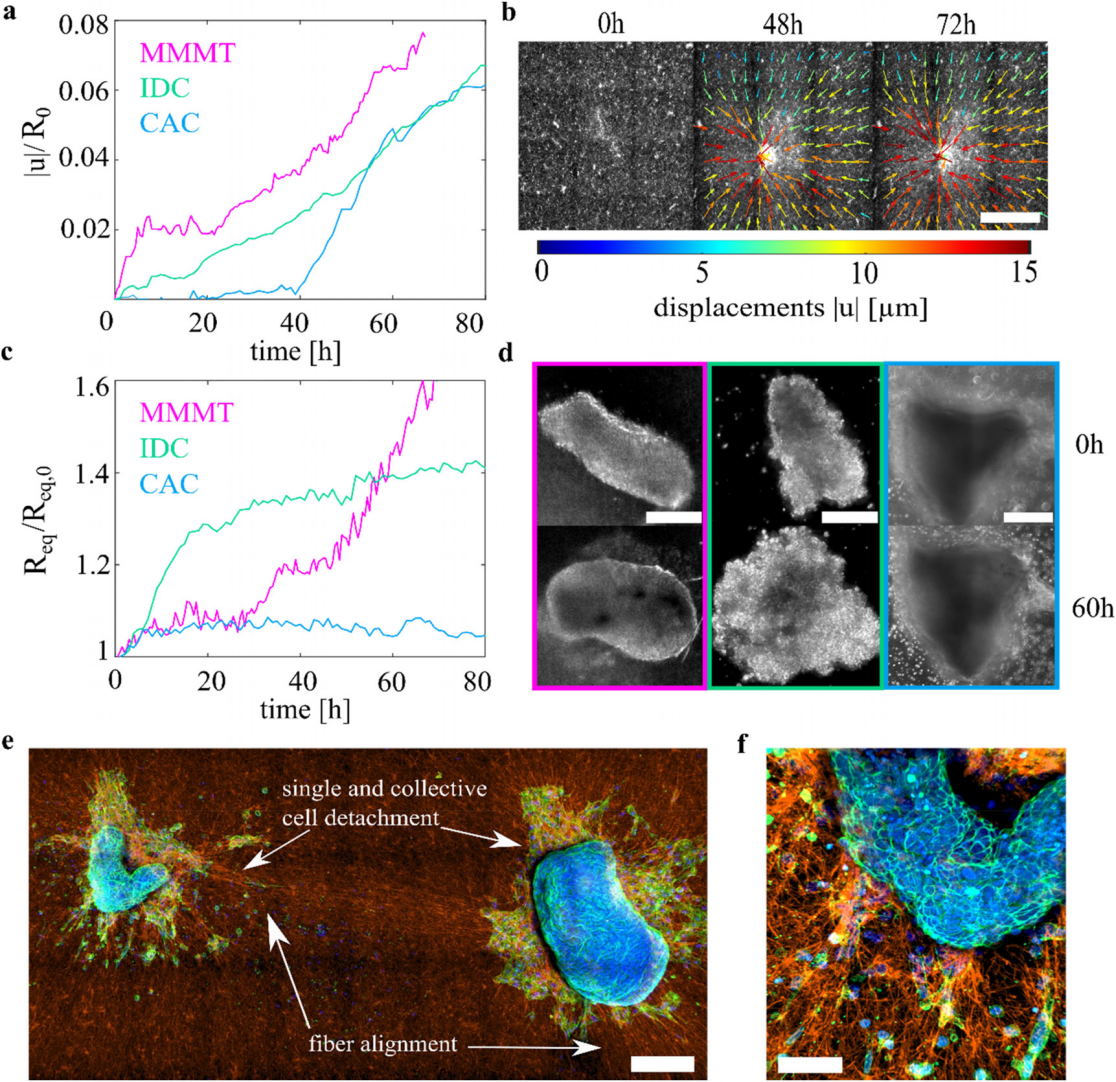
Primary tumor explants contract the collagen network and show not only (single) cell escape but also signs of an active tissue surface tension. (a) Mean normalized collagen displacements induced by three different primary tumor explants. Collagen displacements are averaged over a square region of interest with a side length three times the initial equivalent tumor radius. MMMT = malignant mixed Mullerian tumor, CAC = cervical adenocarcinoma, and IDC = invasive ductal carcinoma. (b) Time course of collagen displacements created by the IDC over time. The strongest displacements are detected below the tumor piece. Scalebar: 500 *μ*m. (c) Normalized equivalent radius R_eq_/R_eq,0_ over time as a measure of cell escape in the different primary tumor explants. (d) Phase contrast images of the three tumor pieces at the beginning of the experiment and after 60 h. Pink: MMMT, green: IDC, and blue: CAC. The field of view is cropped to their corresponding region of interest over which the displacements are averaged. Scalebars: 200, 100, and 200 *μ*m. **e** Fixed and stained MMMT tumor pieces on a 1.5 g/l collagen network. Collective and single cell escape and a smooth tumor explant boundary can be observed. The tumor explants create long-range collagen displacements, forming strong fiber alignment between the pieces. Scalebar 200 *μ*m. (f) Zoomed-in image of the smaller MMMT piece seen in figure (e) on collagen. Collective cortical actin rim and single-cell escape can be seen. Scalebar 100 *μ*m.

To investigate the correlation of the different contractility modes and tumor aggressiveness further, patient-derived tumor explants from breast and cervix are used. The vital primary tumor explants are received directly after surgery. Three (out of six) explants could be cultivated and analyzed. The investigated cancer samples are pathologically classified as malignant mixed mullerian tumor (MMMT), which is a very aggressive cervix tumor containing both carcinoma and sarcoma cells,^44,45^ a cervical adenocarcinoma (CAC), and an invasive ductal carcinoma (IDC) as a breast tumor sample. Again, collagen deformations and radial alignment of the network in the vicinity of the tumor pieces can be observed [see Figs. 3(b)–3(e)].

As described in the "Collagen displacements and cell escape from multicellular spheroids correlate with their aggressiveness" section, the detected collagen displacements are normalized to the equivalent radius of the initial tumor piece area, which had the following values: $R_{0,\mathrm{IDC}}=153$, $R_{0,\mathrm{MMMT}}=206,$ and $R_{0,\mathrm{CAC}}=253\;\mu m$. Because the used tumor pieces vary in their size, the region of interest over which the mean cumulative collagen displacements |u| are calculated is adjusted to a square with a side length of three times the corresponding $R_{0}$. The respective regions of interest are shown for each tumor explant in Fig. 3(d). The MMMT creates the strongest collagen displacements during the observation time, especially during the first 5 h [pink line in Fig. 3(a)]. The CAC only starts to contract the network after 40 h, which indicates that the tumor explant was not strongly attached to the collagen network before. After 40 h, a steeper increase until 70 h is observed, which points toward strong traction forces of the tumor piece during that time. Afterward, the displacements decline again [see blue line in Fig. 3(a)]. The IDC contracts the network on a more continuous level and ends up with a displacement strength comparable to the CAC after 80 h [see green line in Fig. 3(a)]. As a second criterion for the aggressive potential, the spreading behavior of the different cancer explants is evaluated by calculating the normalized equivalent radius R_eq_ of the tumor area for each time point. We found that the MMMT piece shows the strongest spreading throughout the observation time [pink line in Fig. 3(c)]. After 40 h, R_eq_ increases faster than before, which correlates with the onset of pronounced cell escape from the bulk tumor explant and invasion into the collagen network (see Video S4, supplementary material). In contrast to that, the IDC spreading levels off after 20 h [see Fig. 3(c), green line]. The CAC piece displays only little spreading on the collagen network as the blue line in Fig. 3(c) shows. To achieve a coherent understanding, we also assess the boundary texture of the tumor pieces.^46^ The IDC explant does not show any smoothing of the boundaries as would be caused by active tissue surface tension and bulk contraction. Instead, the boundary keeps being rough and numerous cell motion throughout the whole sample (see supplementary material Video S5) can be seen, suggesting a low tissue surface tension. Not much single-cell migration into the ECM is observed in the case of the IDC, and neither in the case of CAC. However, the CAC shows a distinct behavior of activity as the whole piece starts to contract during the measurement time and forms a smooth boundary (supplementary material Video S6). Both suggest the presence of an active tissue surface tension and thus a higher energy barrier, which keeps the cells inside the tumor explant. In comparison, single cell escape and collective invasion can be observed for the MMMT sample, which is also reflected in the course of the normalized equivalent radius $R_{\mathrm{eq}}$ as mentioned above. Here, the strongest interaction with the ECM of all three tumor explants can be seen. Interestingly, a smooth and sharp boundary as well as a contraction of the bulk tumor piece can be observed here, but in this case in a pulsation motion (supplementary material Video S4), which indicates an active tissue surface tension. The existence of both—collective and single cell escape into the ECM and an active tissue surface tension—reflects the high heterogeneity of real tumors. They most probably consist of cells with different properties and phenotypes. With that, a subpopulation with a high stress fiber-based contractility could escape into the ECM, while another subpopulation can generate a collective actin structure and a smooth boundary.^46,47^ A similar behavior is seen for a second piece of the MMMT explant on the same collagen substrate [see left in Fig. 3(d)]. Between both pieces, long-range collagen fiber alignment ranging over roughly 1 mm is detected. For the MMMT patient-derived tumor explants placed on ECM, we find a negative correlation between cell escape and the regions with strong ECM displacements with a correlation coefficient of −0.5980. The strongest displacements of the ECM are generated in regions of the explant boundary where no cells escape and the tumor piece contracts. Since the tumor explant most likely contains a mixture of epithelial-like cells with cortical contractility and mesenchymal-like cells without a pronounced contractile cortex,^19,48^ we expect that boundary regions that cause strong ECM deformations are rich in epithelial-like cells and hinder cell escape by generating a strong tissue surface tension, similar to MCF-10A spheroids. Further details can be found in the supplement (Fig. S4). Based on our results with the breast cancer cell panel, the tumor explants can be classified in terms of their aggressiveness. The MMMT explant shows the most robust and most persistent deformations of the ECM as well as the most pronounced spreading of single cells and cell clusters into it. It is thus classified as the most aggressive, which agrees with the clinical finding that this tumor entity is highly aggressive and has a poor prognosis.^44,45,49,50^ The CAC displays a smooth boundary and an overall contraction. This implies that enough cells within the tumor explant have high cortical contractility and the presence of an active tissue surface tension to trigger this behavior. In contrast, signs of active tissue surface tension cannot be seen in the case of the IDC explant. Thus, the explant is expected to contain fewer cancer cells of an epithelial phenotype that can generate a high tissue surface tension through cortical contractility. According to the previous experiments with the cell lines, a smooth surface pointing to a high cortical tension hinders cell escape and can consequently be associated with less formation of metastases. As the IDC can still create strong collagen displacements, it is ranked here to be more aggressive than the CAC piece.

In the clinical context, the aggressiveness of real tumors is classified by the grading, TNM staging and molecular factors.^51,52^ Each tumor can thus be classified individually on different scales. The grading describes how dedifferentiated and disordered the cancer cells in the tumor are, meaning, in the case of carcinomas, how much the tumor structure differs from well-ordered epithelium.^53^ The TNM staging ranks tumors according to their size or how much they infiltrate adjacent tissues (T) if the lymph nodes are invaded (N), and if the tumor forms metastases (M).^54^ As the tumor explants originate from different entities, we use the grading of each individual tumor to compare their real aggressiveness order underneath each other. The malignant mixed Mullerian tumor (MMMT) is graded G3, the invasive ductal carcinoma (IDC) is graded G2, and the cervical adenocarcinoma (CAC) is graded G1 (see also Table I). The tumor aggressiveness that we obtained from our assay correlates well with the clinical grading of the tumor samples. However, at this point, this finding could be incidental as tumors are highly heterogeneous, and we observed only small sections of a far larger tumor in each case.

**TABLE I.** Biophysical parameters and characteristics of the used cell lines and primary tumor pieces. The first two columns correspond to the axes of the malignancy phase diagram. The cortical contractility of single suspended cells was not measured for the primary tumor pieces. G: Grading, T: tumor size, N: lymph node invasion, and M: metastases. All tumors had lymphovascular invasion (L1).

	$\frac{d}{\mathrm{dt}}$|u| (*μ*m/h)	$\frac{d}{\mathrm{dt}}$ A/A_0_ (1/h)	σ_int_ (Pa)	Morphology	Malignancy
MCF-10A	0.20	0	1.09	Epithelial	Benign
MDA-MB-436	0.24	0.031	0.15	Mesenchymal	Moderately invasive
MDA-MB-231	0.82	0.162	0.39	Mesenchymal	Highly invasive
CAC	0.33	0.033	⋯	Heterogeneous	G1 pT2b pN0 pM1 L1
IDC	0.35	0.044	⋯	Heterogeneous	G2, T2 cN0 pM1 L1
MMMT	0.37	0.037	⋯	Heterogeneous	G3, pT2b pN0 pM0 L1

### Number of highly cortical contractile cells decreases with tumor aggressiveness in primary tumor explants

The experiments with primary tumor explants on collagen matrices revealed distinctive cellular spreading patterns as well as specific interactions of the whole tumor explants with the collagen matrix as ECM model. The well-characterized properties of the breast cancer cell panel with varying aggressiveness link the different spreading behavior of the explants to the cortical contractility of single cells and the ability to form collective actin structures. Cancer cells are typically associated with a more mesenchymal phenotype through the epithelial-to-mesenchymal transition and have a softer actin cortex during interphase.^33,55,56^ To gain more insight into this topic, an additional set of measurements with primary tumor explants, dissociated into single cells, is performed. Their mechanical properties are measured again with the optical stretcher to show that primary tumors also contain cells of a more epithelial phenotype with a high cortical contractility. Cells of nine tumor explants from four different cancer types and entities are measured, namely, three cervical adenocarcinomas (CAC), two cervical squamous carcinomas (CSCC), two mixed Mullerian carcinomas (MMMT), and two invasive ductal carcinomas (IDC).

**FIG. 4.**
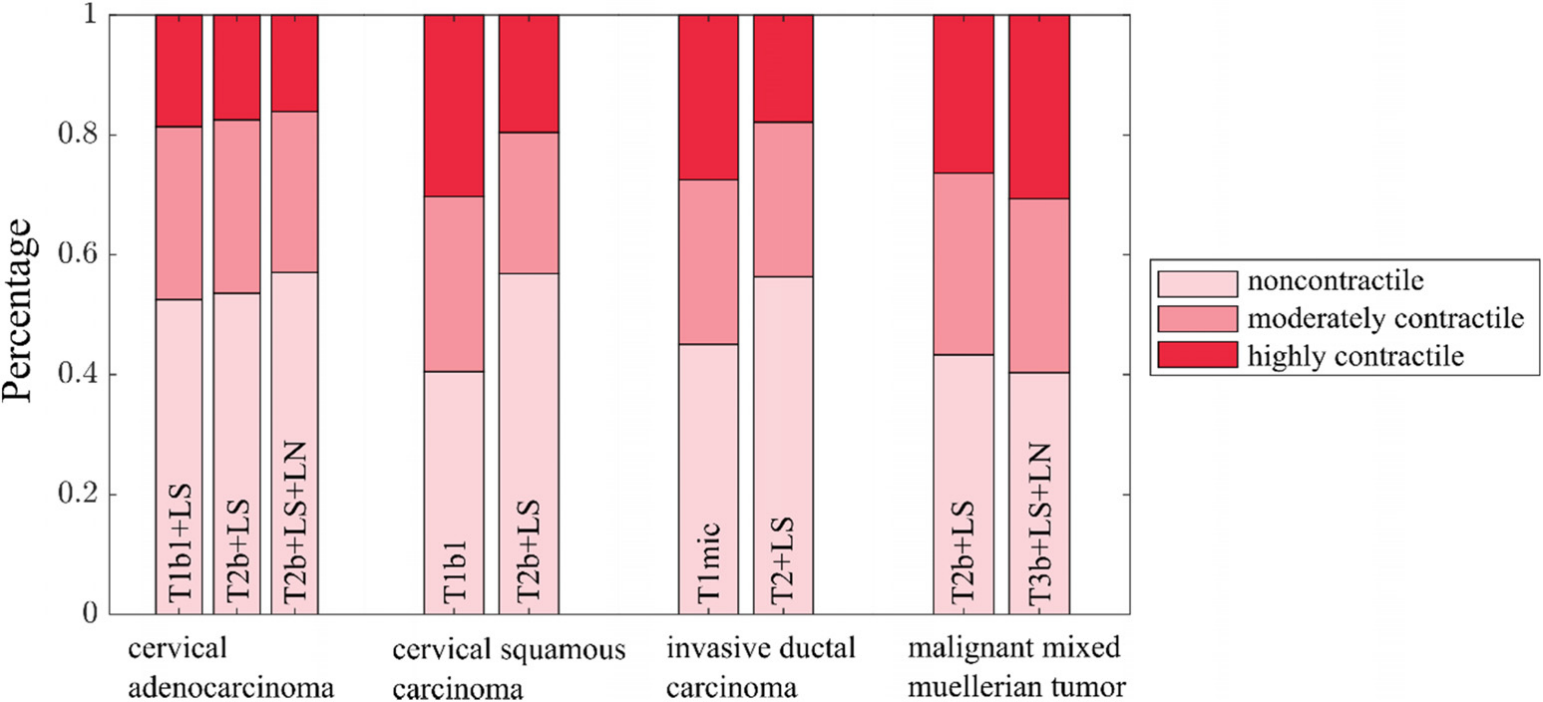
Contractility distribution of single cells from patient-derived tumor explants. Percentage of non-contractile (σ_int_ < 0.1 Pa), moderately contractile (0.1 Pa < σ_int_ < 0.8 Pa), and highly contractile (0.8 Pa < σ_int_) cancer cells from different tumor entities measured with the optical stretcher. The corresponding TNM staging is written inside the bar plots and increases for each tumor entity from left to right. The amount of weakly contractile cells increases during carcinoma progression, while the amount of highly contractile cell decreases (see also Table II).

Unfortunately, typically the tumor material obtained from surgery was not enough to perform both collagen and optical stretcher measurements. Only for one MMMT explant, a complete dataset could be obtained. The cells of each sample are assigned into the fraction of highly contractile, moderately contractile, and non-contractile cells to show that the amount of highly contractile cells decreases while the amount of non-contractile cells increases with tumor progression. A cell is considered *highly contractile* when the contractile stress is opposing the pulling stress of the optical stretcher (σ_int_ > 0.8 Pa) during the stretch phase and as *non-contractile* when the contractile stress is less than 0.1 Pa.^19^ The group of *moderately contractile* cells falls in between these two groups. Since the tumor grading was mainly the same within the compared tumor samples and only tumors within the same entity are compared to each other (in contrast to the previous collagen experiments), the TNM staging^54^ is used here to differentiate the aggressiveness of the measured tumor explants (see also Fig. 4). In the TNM staging, the parameter T reflects the local tumor extend as defined by size and invasion into adjacent tissues. The parameters N and M are binary and indicate if the lymph nodes are invaded (N) or if the tumor forms metastases (M). To further stratify the aggressiveness, the invasion of nearby lymph vessels (L) is further included. The exact staging and grading for each tumor explant can be found in Table II.

**TABLE II.** n: number of measured cells, σ_int_ > 0.8 Pa highly contractile cells, σ_int_ < 0.1 Pa non-contractile cells, G: grading, T: tumor size, N: lymph node invasion, and M: metastases. All tumors had lymphovascular invasion (L1) and none formed metastases (M0), so this parameter is not shown in the table.

	n	σ_int_ > 0.8 Pa (%)	σ_int_ < 0.1 Pa (%)	Grading	T-staging	Lymph node invasion
CAC1	446	17.79	53.59	G3	T2b	N0
CAC2	880	18.64	52.50	G3	T2	N1
CAC3	261	16.09	57.09	G3	T2b	N1
CSCC1	264	30.30	40.53	G2	T1b1	N0
CSCC2	577	19.58	56.84	G3	T2b	N0
MMMT1	773	26.39	43.34	G3	T2b	N0
MMMT2	741	30.63	40.35	G3	T3b	N1
IDC1	621	17.87	56.36	G2	T2	N0
IDC2	182	27.47	45.05	G2	T1mic	N0

The cervical adenocarcinomas (CAC), the cervical squamous carcinomas (CSCC), and the invasive ductal carcinomas (IDC) follow the same trend: With increasing aggressiveness, the number of highly contractile cells decreases while the number of non-contractile cells increases. This supports our hypothesis that during cancer progression, the number of cells that lose their cortical contractility and transform to a mesenchymal phenotype with a dominant stress fiber-based contractility increases. As confirmed by the experiments with the cell lines, the loss of cortical contractility appears to be an essential feature during cancer cell escape from the initial cell cluster.

An exception from this trend is the opposite behavior of the two malignant mixed Mullerian tumors (MMMT), in which the number of highly contractile cells in the optical stretcher increases, and the percentage of non-contractile cells decreases with tumor progression. However, MMMTs are tumors with a p53 mutation and develop a sarcomatous component, which is why they are named “mixed.” Cells in this component are characterized by a mesenchymal phenotype and a high affinity to the ECM [see also Figs. 3(d) and 3(e)]. This characteristic makes them highly aggressive and different from normal carcinomas. The size of the sarcomatous part and their specific cell properties differ for each tumor and reach from fibrosarcoma to osteosarcoma features.^57,58^ Therefore, the concept of decreasing cortical contractility cannot simply be transferred onto these tumors, which is why an opposing behavior might be observed here. In pure carcinoma, such as CAC, CSCC, and IDC, the EMT is an essential element in tumor development.^59–61^ This is reflected in the cortical contractility measurements with the optical stretcher. Here, our results reveal decreasing cortical contractility during cancer progression, thereby lowering the energy barrier for cells to escape the original cell cluster and thus promoting a higher aggressiveness.

## DISCUSSION

**FIG. 5.**
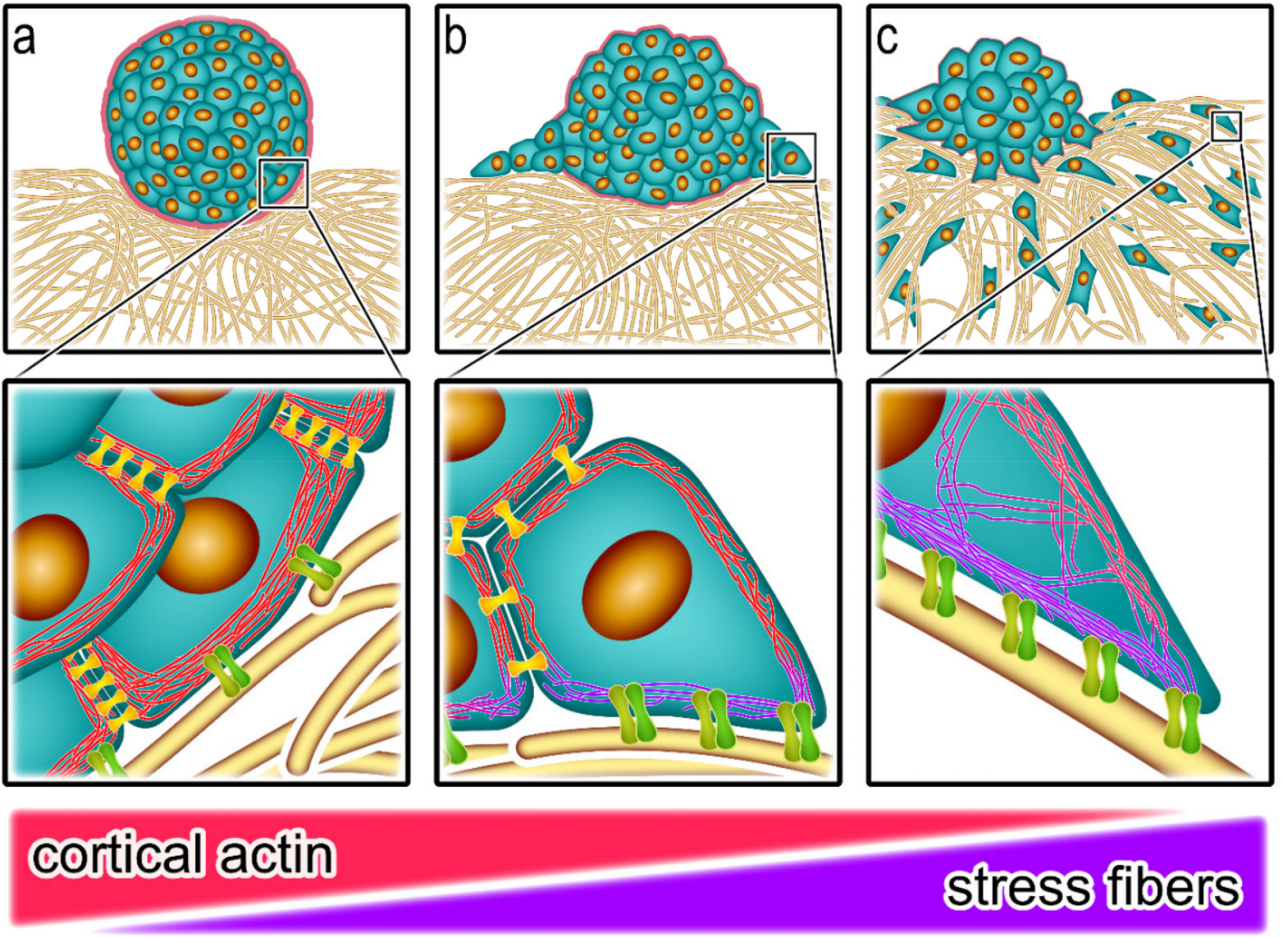
Effects of different contractility modes on cell interaction with the ECM and cancer cell escape. (a) stress-fiber contractility < cortical contractility: the cells form a collective cortical actin rim across the cells at the spheroid boundary (red). This active contractile shell around the cell aggregate generates enough tension to hold the cells inside the spheroid. The cells still interact with the ECM and contract the collagen network in contact with the cortical rim, which results in an accumulation and alignment of collagen fibers around the spheroid. (MCF-10A) (b) stress-fiber contractility ≈ cortical contractility: The cell aggregate spreads on the collagen substrate in a wetting process. Cells can migrate on the collagen surface, but only minor invasion into the ECM is possible. The invading cancer cells switch their dominant actin architecture from a collective cortical mode (red), which is formed via cell–cell contacts (yellow) to stress-fiber contractility (purple). (MDA-MB-436) (c) stress-fiber contractility > cortical contractility: cancer cells dissociate from the cell aggregate in an evaporation-like process. A quasi-complete dissolution of the spheroid into the ECM is achieved. The invading cells adhere to the ECM via integrins (green) and form stress-fibers as their dominant actin structure (purple) to pull themselves into the ECM. (MDA-MB-231).

The important role of the interaction between tumors and the ECM has been demonstrated widely in the last few years. Solid tumors trigger their own form of fibrosis, which forms a unique extracellular matrix that influences the tumor microbiome and protects against immune reactions. Collagen, particularly unstructured, plays a protective role for cancer cells by altering the cellular metabolic status to a more glycolytic state which hinders drug delivery.^22,24,62^ In this context, desmoplasia and collagen biomarkers have been linked to relapse and death in cancer patients.^24,25^ The stiffness of the ECM affects the traction forces of the cells and influences the nuclear deformation as well as their gene expression.^63–66^ The traction forces further modulate the tumor microenvironment and the interactions between cancer cells and stromal cells.^67–69^ The present study connects local ECM remodeling and stiffening that is created by cell spheroids or primary tumor explants to cancer cell escape and invasion. Our results suggest that local cell escape is determined by the interplay between different contractility modes of single cells. There is a multitude of factors that influence cancer cell invasion into the ECM. Since our focus is the role of cell contractility factors such as cell–cell or cell–ECM adhesion are highly relevant since adhesion receptors directly connect to the cytoskeleton. While factors such as MMP activity do not directly relate to our focus. Epithelial cells with a high cortical contractility and strong cell–cell adhesions, such as myoepithelial and luminal epithelial cells of the milk ducts in the breast, form a strong collective actin rim at their cell boundary, which is supported by an according assembly of cadherin junctions as intercellular connections.^70^ The formation of cell–cell junctions induces a downregulation of the cortical actin at the junction site but strengthens the tension at the non-contact areas, thus increasing the overall tissue surface tension.^19,71–77^ The high tension compacts the cell aggregate. Consequently, the cells are in a jammed, non-motile state and stay inside the cell cluster.^6,8^ The collective cortical actin rim acts as an energy barrier that cells must overcome to leave the spheroid. Despite the shielding effect of the collective actin rim, epithelial cell clusters still interact with the surrounding ECM and form contacts through focal adhesions, which can be seen by the collagen displacements and an aligned and condensed collagen network around the cell cluster [see Fig. 5(a)]. The strength of the interaction with the ECM is insufficient to overcome the generated active tissue surface tension and thus for effective cancer cell invasion. The increase in tissue surface tension through cortical contractility holds the cells back in MCF-10A spheroids, while the lower cortical contractility in the other spheroids decreases tissue surface tension to permit cell escape. This process can be seen as a competition between the cortical contractility on one side and the stress fiber-based contractility on the other side. Cells with lower cortical contractility and more pronounced stress fiber contractility escape more easily from the original cell cluster. Here, the initial energy barrier is thought to be lower, and the interaction with the ECM is stronger.

As soon as the interaction with the ECM and thus the generated traction forces are high enough to overcome this energy barrier, cells can leave the cell cluster. Cells with strong and dominant stress-fiber contractility, such as MDA-MB-231 cells, individually leave the cluster as single cells, which can be interpreted as an evaporation process [see Fig. 5(c)]. If the interaction strength with the ECM is weaker, we observe more of an active collective wetting transition, where the cell aggregate spreads similarly to a monolayer [see Fig. 5(b)]. Both migration modes have been described before on two-dimensional substrates.^78,79^ We now identified similar behaviors in a three-dimensional collagen environment both for multicellular spheroids and for patient-derived tumor explants, which resemble more the ECM in the tumor microenvironment.^79,80^ Our results suggest that cells of a more epithelial phenotype with a high cortical contractility stabilize cell aggregates by generating a high tissue surface tension, which is indicated by the smooth spheroid surface and highly elongated cells at the boundary. This applies to MCF-10A clusters. In our experiments, the highly invasive MDA-MB-231 cells could not form stable aggregates, even though some cortical contractile behavior is observed in the optical stretcher. However, we also observed a strong stress fiber-based interaction with the ECM. This interaction is much stronger than the weak cortical contractility and dominates the overall cellular behavior. MDA-MB-436 cells have an even lower cortical contractility but also a low stress fiber-based contractility, so none of them trumps the other, and we observe weak behavior from both contractility modes. *Vignaud *et al.** showed that stress-fibers and a contractile actin cortex are not independent from each other, which agrees with our findings.^14^ MDA-MB-436 spheroids show a wetting-like behavior on the collagen substrates. As recently reported, the wetting of cell aggregates is an active process where the more cohesive aggregate is coated by a monolayer of less cohesive cells, driven by active cortical tension or an increase in the internal pressure through elastocapillary effects on soft substrates.^79,81^ We expect that we have a similar situation in our experiments where cells are wetting an adhesive collagen sheet. Thereby, we would like to stress that cells are hold back from wetting not by cell–cell adhesion but by active tissue surface tension generated by cortical contractility. The classical differential adhesion hypothesis as original explanation how tissue surface tension is generated attributes stable cell boundaries to differences in cell–cell adhesion,^82^ while the extended differential adhesion hypothesis added cell contractility as a contributing factor to tissue surface tension.^83,84^ Our experiments suggest that similar mechanisms play a role in the process of cancer cell escape into the ECM. We hypothesize that different contractility modes and not differential adhesion are the predominant factor stabilizing cell cluster boundaries found in solid tumors as already proposed in Ref. 85, and that switching from predominantly epithelial cortical to predominantly mesenchymal stress fiber contractility fosters cancer cell escape This also implies a decrease in the overall tissue surface tension, which qualitatively becomes visible by a higher roughness of the tissue or rather spheroid boundary.^10,19,84^ This relates to our breast cancer panel, as the boundary becomes rougher and less sharp with increasing cell invasion into the ECM. Previous studies^10,19,86^ that analyzed cell segregation in mixtures of the cell lines used here have found that the MCF-10A cells have the highest cohesion (i.e., tissue surface tension), MDA-MB-436 cells show lower cohesion, and MDA-MB-231 cells the lowest cohesion. This agrees with our findings that MCF-10A spheroids are the most stable, MDA-MB-436 spheroids are less stable, and MDA-MB-231 cells have to be stabilized by Matrigel to form spheroids.

With our assay, we found a coarse-grained predictor for the intricate mechanism if cancer cell cluster boundaries become unstable and permit cancer cell escape. The competition between these two contractility modes is a conclusive factor to induce cancer cell escape from cell clusters as an early event in metastasis. Since carcinomas do not undergo a complete EMT and cancer cells show a mixed epithelial and mesenchymal phenotype, it is not the presence of a single molecular marker, such as cadherins or integrins, that unambiguously indicates cancer cell escape.^21,87^ Instead, active cell properties that are both based on acto-myosin contraction and can be converted into each other during cancer progression are used to determine the risk of cancer cell escape.^16,17,88^ This implies that the “switch” between a stable, defined cell boundary to the ECM and an unstable boundary that permits cells to invade the ECM is the competitive interplay between these two different contractility modes.

This hypothesis is further strengthened by measurements with primary tumor explants, either on the collagen assay or with the optical stretcher. On the collagen networks, the primary tumor explants show a wide spectrum of epithelial effects, like a sharp and smooth boundary, and mesenchymal effects, such as single and collective cell escape. This is most likely attributed to different cell populations that are present in the primary tumors which have an either more epithelial or mesenchymal phenotype. The optical stretcher measurements confirm these observations. The cortical contractility, which is a property associated with epithelial cells, decreases during tumor progression while the amount of cells with a low to vanishing cortical contractility increases. Future studies will have to investigate whether this is due to inherent tumor heterogeneity, being composed of both epithelial-like and mesenchymal-like cells or even intermediates,^21,87,89^ or of hard and soft cells.^47,90^ They should determine further how our potential diagnostic criteria perform with respect to existing diagnostic approaches and if they provide complementary, additional prognostic information. In particular, it will be necessary to correlate the properties of primary tumor pieces with distant metastatic risk to investigate their essential role in the metastatic cascade.

## METHODS

### Cell lines

MCF-10A cells are cultured in a medium composed of DMEM/Hams F12 medium with L-glutamine (Catalog No. E15-813, PAA Laboratories GmbH, Austria) supplemented with 5% horse serum (Catalog No. A15-151, PAA), 20 ng/l human EGF (Catalog No. E9644, Sigma-Aldrich), 10 mg/l insulin (Catalog No. I9278, Sigma-Aldrich), 100 ng/l cholera toxin (Catalog No. C8052, Sigma-Aldrich), 500 ng/l hydrocortisone (Catalog No.H0888, Sigma-Aldrich), and 100 U/ml penicillin/streptomycin (Catalog No. P11-010, PAA). MDA-MB-436 and MDA-MB-231GFP cells are cultured in a medium composed of DMEM containing 4.5 g/l glucose and L-glutamine, but without sodium pyruvate (Catalog No. E15-810, PAA) supplemented with 10% fetal bovine serum (Catalog No. A15-151, PAA) and 100 U/ml penicillin/streptomycin. We only used MDA-MB-231GFP cells and refer to them for simplicity in the following only as MDA-MB-231 cells. All cells are cultured at 37 °C and 5% CO_2_, passaged at 80% confluency. The medium is changed every 2–3 days. For passaging, the cells are two times washed with PBS (phosphate buffer solution) and then detached with 0.025% (wt./vol.) trypsin and 0.011 (wt./vol.) ethylenediamine tetra-acetic acid (Catalog No. L11-004, PAA).

### Formation of multicellular spheroids

For MCF-10A and MDA-MB-436 spheroids, approximately 1000 cells were seeded into 96 low-adhesion U-wells (Sigma, BR781900) and cultivated inside for 48 h. MDA-MB-231 cells do not self-assemble into stable aggregates in 48 h. Therefore, 3.5% Matrigel^TM^ (Corning TM 354234) were added to 250 cells and seeded into low-adhesive U-wells. With this approach, aggregates with a comparable size and shape to the other spheroids were obtained. MDA-MB-231 cells do not generate a high surface tension since these cells neither generate high cell–cell adhesion nor high cortical contractility. Thus, we had to add Matrigel as additional cellular glue to obtain spheroids. For MDA-MB-436 cells, cell–cell adhesion alone is strong enough to form stable spheroids. Additional Matrigel^TM^ did not result in more compact aggregates for MDA-MB-436 cells.

### Preparation of primary tumor samples

Primary tumor samples were directly obtained from surgery. Fresh samples are put into DMEM and cut into small pieces (100–500 *μ*m) with a scalpel. Afterward the tumor pieces were placed on the collagen networks with a pipette. For the experiments, the samples were cultured in DMEM.

### Fixation, staining and imaging of cellular samples

All samples were fixed with 4 (wt. %/vol. %) paraformaldehyde in PBS for 30 min. Then, they were washed 2–3 times with PBS with a 5 min waiting time between each washing step. Then, the samples were placed in 1% Triton-X overnight and washed afterward with PBS again. All fixed samples were stained with 1 *μ*M Alexa-Flour488 Phalloidin (Thermo-Fisher, Waltham) and 2 mg/ml Hoechst 34580 (Invitrogen, H21486). Since imaging through the collagen sheet is impossible with good quality, a circular piece (d = 8 mm) of the collagen network around the cell aggregate in question is punched out and then flipped upside down in a new well of a 24 *μ*-well plate. Through this approach, the spheroid directly faces the objective of the inverted microscope and can be easily imaged. However, the punch out process can lead to disturbances in the collagen matrix and tension relaxation^13^ may occur, so that the collagen structure including alignment may not be representative of the situation during the vital measurements.

### Preparation and staining of collagen networks

Hydrogels with a final concentration of 1.5 g/l type I collagen were polymerized from a 1:2 mixture of rat-tail and bovine skin collagen as described previously.^91^ In brief, for 1 ml of the final hydrogel, 125 *μ*l type I rat-tail collagen (Collagen R solution 0.4%, Lot# 190193, Serva Electrophoresis, Heidelberg, Germany) and 250 *μ*l type I bovine skin collagen (Collagen G solution 0.4%, Lot# 0235G, Biochrom GmbH, Berlin, Germany) were dissolved in 625 *μ*l of a 1 M phosphate-buffered solution (168 *μ*l Na_2_HPO_4_, 32 *μ*l NaH_2_PO_4_, H_2_O and 425 *μ*l doubled distilled H_2_O).

To prevent premature polymerization during the preparation, the process was carried out on the ice, and the collagen solution was immediately transferred into the sample carrier of choice and incubated for 90 min at 37 °C and 95% humidity. Afterward, the polymerized collagen gels were washed three times with PBS and stained with 1 mg/ml TAMRA-SE overnight. Then the collagen networks were washed three times with PBS and incubated with the desired cell culture medium. 3.0 g/l collagen gels were prepared in a similar way but with adapted parameters.

### Live observation of cell spreading in collagen networks

In each well of a 24 well *μ*-plate (ibidi, Martinsried, Germany), 250 *μ*l collagen was polymerized as described above. After adequate incubation with cell culture medium, a single spheroid was transferred on top of each collagen gel. The plate was immediately brought onto an inverted Zeiss Axio Observer microscope setup with incubation chamber 37 °C, 5% CO_2_) to start the measurement. The experiments were recorded over the course of 60 h every 15 min with an EC Plan-Neofluar M27 5×/0.16 objective and an EC-Plan-Neofluar M27 2.5×/0.12 objective in one image plane. MCF-10A, MDA-MB-436, and MDA-MB-231 spheroids were stained with SiR-DNA at a concentration of 0.2 *μ*M. The temporal development of the migration behavior is tracked automatically with a self-written MATLAB script, which uses the fluorescent signal of the cell nuclei.

Quantification of the collagen displacements was performed as follows. The collagen displacements are detected from the brightfield images with a self-written MATLAB code based on a PIV (particle image velocimetry) algorithm. The displacements are added up at each point per time step, so that cumulative displacements are obtained. The displacements are then averaged over the field of view. As larger spheroids create larger displacements, the cumulative mean displacements are normalized to the initial spheroid radius, which is detected by a self-written MATLAB script.

### Quantification of cell escape and spreading

The spreading behavior is determined over the area covered with cells, which is detected over the fluorescent signal. For the analysis of the primary tumor pieces, we detected the tumor piece boundary by hand using *ImageJ* over the complete observation time. We chose this detection method for the primary samples as the image quality in these samples did not always allow for the automatic detection. Then, the square root over π is calculated to obtain the average equivalent spreading radius, which is then normalized to the initial spheroid or rather equivalent tumor piece radius.

### Optical stretcher measurements

For the optical stretcher measurements, cells were detached from the cell flask according to the protocol above. After the centrifugation process, the cell pellet is resuspended in 1 ml of the corresponding medium. For the measurements, the cells are flushed into the microfluidic optical stretcher system. Phase-contrast images of suspended cells were recorded by an Andor Zyla 5.5 sCMOS camera mounted on an inverted Zeiss Axio Observer Z1 and an LD Plan-Neofluar 63×/0.75 Korr Ph 2 M27 objective.

Single cells were trapped with a laser power of 100 mW for 1 s, then stretched with 875 mW for 5 s, and then trapped again with 100 mW for 2 s. Further details on the method can be found in Ref. 19.

### Quantification of the optical stretcher measurements

To quantify the passive and active viscoelastic cell properties, a SLS (standard linear solid) model in Kelvin–Voigt representation is fitted on each cell's deformation curve. The model consists of a spring and dashpot in parallel and an additional spring in series, which corresponds to the active pretension of the cell. The active cortical contractility is modeled through an internal linear stress (σ_int_) opposing the external stresses applied by the lasers. The resulting effective stress (σ_eff_) is larger than zero for non-contractile cells and smaller than zero if the cortical contractility is larger than the external stress.

### Cell segmentation and shape analysis

To enhance the image quality, several preprocessing steps are implemented. This included background subtraction, noise reduction, and contrast adjustment, which are essential to improve the accuracy of subsequent measurements by mitigating potential artifacts and enhancing the clarity of cellular features. For the initial cell shape analysis, *CellPose*^92,93^ an advanced deep learning-based algorithm designed for cell segmentation is employed. In cases where *CellPose* did not adequately identify cell shapes, manual interventions are performed to supplement missing cell outlines. The resultant cell outlines are further analyzed with MATLAB using a custom-developed algorithm. The algorithm calculates the cell shape index p_0_ = $P_{0}/\sqrt{A_{0}}$ with P_0_ as the cell perimeter and A_0_ as the cell area.^7^

### Preparation of primary tumor samples for optical stretcher measurements

Vital single cells were isolated from tumor pieces using a Miltenyi Tumor Dissociation Kit, human (Cat. No. 130-095-929, Miltenyi Biotec, Bergisch Gladbach, Germany), employing a Miltenyi gentleMACS Dissociator (Cat. No. 130-093-235, Miltenyi Biotec), and Miltenyi gentleMACS C Tubes (Cat. No. 130-093-237, Miltenyi Biotec). Dissociation was executed according to the protocols provided by the supplier, with the exception of performing two straining steps instead of one after dissociation and reducing centrifugation force to 200 g. Cancer cells were separated from other residual cells employing magnetic bead sorting, using CD235a MicroBeads, human (Cat. No. 130-050-501, Miltenyi Biotec) and Anti-Fibroblast MicroBeads, human (Cat. No. 130-050-601, Miltenyi Biotec), following the protocol provided by the supplier except for reducing centrifugation force to 200 g.

### Mechanical measurements with the AFM

AFM measurements were done to prove that the pulling forces of the cells lead to strain-stiffening effects in the surrounding collagen network. The collagen surface was tested with a Zeiss AxioZoom.V16 microscope (Zeiss, Oberkochen, Germany) equipped with a JPK BioAFM HybridStage (Bruker Nano GmbH, Berlin, Germany). A Pointprobe-CONT cantilever (Nanoworld, Neuchâtel, Switzerland) was modified with a polystyrene bead (45 *μ*m diameter) to increase the contact area between sample and indenter. Indentations were performed at various positions with a maximum set force of 5 nN while the sample was fully submerged in cell-culture medium and maintained at a constant temperature of 37 °C. Force-indentation curves were fitted with a Hertz model to create an elasticity map, showing Young's modulus in the vicinity of the spheroid. Figure S5 demonstrates exemplary for an MCF-10A spheroid that the collagen stiffness increases closer to the edge of the spheroid.

## SUPPLEMENTARY MATERIAL

See the supplementary material for measurements on 3.0 g/l collagen, details on the correlation between the collagen displacements and the cell spreading and their distribution, AFM measurement, and classification of the detection of the collagen displacements in different z-planes.

## Data Availability

The data that support the findings of this study are available from the corresponding author upon reasonable request.
